# Comparative Plastomes and Phylogenetic Analysis of *Cleistogenes* and Closely Related Genera (Poaceae)

**DOI:** 10.3389/fpls.2021.638597

**Published:** 2021-03-25

**Authors:** Rong Wang, Kuan Liu, Xue-Jie Zhang, Wen-Li Chen, Xiao-Jian Qu, Shou-Jin Fan

**Affiliations:** ^1^Shandong Provincial Key Laboratory of Plant Stress Research, College of Life Science, Shandong Normal University, Jinan, China; ^2^State Key Laboratory of Systematic and Evolutionary Botany, Institute of Botany, Chinese Academy of Sciences, Beijing, China

**Keywords:** *Cleistogenes*, comparative genomics, plastome evolution, molecular marker, phylogenetic relationships

## Abstract

*Cleistogenes* (Orininae, Cynodonteae, Chloridoideae, Poaceae) is an ecologically important genus. The phylogenetic placement of *Cleistogenes* and phylogenetic relationships among *Cleistogenes* taxa remain controversial for a long time. To resolve the intra- and inter-generic relationships of *Cleistogenes*, the plastomes of 12 *Cleistogenes* taxa (including 8 species and 4 varieties), one *Orinus* species, 15 *Triodia* species, two *Tripogon* species, and two *Aeluropus* species were included in the present study. All the taxa showed a similar pattern in plastome structure, gene order, gene content, and IR boundaries. The number of simple sequence repeats ranged from 145 (*O. kokonorica*) to 161 (*T. plurinervata* and *T. schinzii*). Moreover, 1,687 repeats were identified in these taxa, including 1,012 forward, 650 palindromic, 24 reverse, and one complement. Codon usage analysis revealed that these plastomes contained 16,633 (*T. stipoides*) to 16,678 (*T. tomentosa*) codons. Sequence divergence analysis among *Cleistogenes* and closely related genera identified five non-coding regions (*trnS-UGA-psbZ*, *rpl32-trnL-UAG*, *trnQ-UUG-psbK*, *trnD-GUC-psbM*, *trnT-GGU-trnE-UUC*). Phylogenetic analysis of complete plastomes indicated that *Cleistogenes* is sister to a clade composed of *Orinus* and *Triodia*, whereas it did not support the sister relationship between the recently proposed subtribe Orininae (*Cleistogenes* and *Orinus*) and *Triodia*. The subtribe Orininae was not supported by our complete plastome data. The split between *Cleistogenes* and *Orinus*-*Triodia* clade go back to 14.01 Ma. Besides, our findings suggested that *C. squarrosa* and *C. songorica* are the successive early diverging groups in the phylogenetic analysis. The other 10 taxa are divided into two groups: a monophyletic group composed of *Cleistogenes* sp. nov. and *C. caespitosa* var. *ramosa* is sister to other eight *Cleistogenes* taxa. *Cleistogenes* was estimated to have experienced rapid divergence within a short period, which could be a major obstacle in resolving phylogenetic relationships within *Cleistogenes*. Collectively, our results provided valuable insights into the phylogenetic study of grass species.

## Introduction

As an ecologically important genus in the grass family (Poaceae) (Liang et al., 2002; Wang et al., 2003), *Cleistogenes* is composed of about 13 species, which are mainly distributed in South Europe to Turkey and eastward through central Asia, China, Pakistan, Northwest India, and Japan (Chen, 1990; Chen et al., 2006; Clayton et al., 2006). A large proportion of such species are found in semi-arid regions, where they are excellent forage grass and sand-binding grass (Shan et al., 2010; Shi, 2011; Tao et al., 2017). It is well documented that *Cleistogenes* has the C_4_ photosynthetic pathway and can adapt to a dry environment (Redmann et al., 1995; Su et al., 2011). Two *Cleistogenes* species including *C. squarrosa* and *C. songorica* are commonly used as plant materials to study grass competition and drought stress (Gao et al., 2005; Niu and Nan, 2017). This genus is remarkable for the cleistogamous spikelets, which often exist in the leaf sheaths to ensure the seed production even under severe conditions. These cleistogamous spikelets usually have fewer florets, which are smaller and narrower lemmas with longer awns compared with the chasmogamous spikelets (Chen et al., 2006).

Several phylogenetic studies have studied *Cleistogenes* and its closely related genera, while the inter-generic relationships remain uncertain. *Cleistogenes* is either classified within Eragrostideae or Cynodonteae based on morphological characters in traditional classification (Tutin and Al, 1980; Tzvelev, 1989; Chen, 1990; Chen et al., 2006). These two tribes are belonging to the subfamily Chloridoideae. *Cleistogenes* has been suggested to be within Cynodonteae in molecular phylogenetic analyses (Peterson et al., 2010; Soreng et al., 2015, 2017). *Cleistogenes* is considered as an incertae sedis genus due to its different placement in ITS, plastid, and combined trees (Peterson et al., 2010; Soreng et al., 2015). Recent studies show that Aeluropodinae (*Aeluropus* and *Odyssea*), Triodiinae (*Triodia*), and Orininae (*Cleistogenes* and *Orinus*) form a monophyletic clade (Peterson et al., 2017; Soreng et al., 2017), while the relationships among them are incongruence. *Cleistogenes* is sister to *Orinus* in an ITS tree, and in a combined tree (Peterson et al., 2010, 2016). The clade composed of *Cleistogenes* and *Orinus* is sister to the clade consisting of Aeluropodinae and Triodiinae in the ITS tree (Peterson et al., 2010) and a successive sister to Aeluropodinae and Triodiinae in the combined tree (Peterson et al., 2016). *Cleistogenes* and *Orinus* are difficult to seperate morphologically, and *Orinus kokonorica* was once described as a *Cleistogenes* species (Hao, 1938). Cleistogamous spikelets are morphological characters used to distinguish *Cleistogenes* from *Orinus* (Chen, 1990; Chen et al., 2006). Based on these studies, Peterson et al. (2016) have proposed a new subtribe Orininae, including *Cleistogenes* and *Orinus*. However, inter-generic relationships of *Cleistogenes* and closely related genera remain poorly resolved in the plastid tree (Peterson et al., 2010). The phylogenetic relationships among *Cleistogenes* and closely related genera are controversial. Moreover, the sister relationship between *Cleistogenes* and *Orinus* was merely moderately supported. Poor phylogenetic resolution is likely due to the lack of informative sites in the selected molecular markers, which appears to be a common problem in multi-locus phylogenetic studies. A robust phylogenetic tree between *Cleistogenes* and closely related genera was urgently need to understand their phylogenetic relationships among *Cleistogenes* and closely related genera.

Only very few studies have studied the intra-generic relationships of *Cleistogenes*. Species delimitation is explored using morphological characters. *Cleistogenes* was previously considered to be invalid because the name is coincident with a technical term. *Kengia* was originally published to replace *Cleistogenes* Keng (Packer, 1960). However, Art. 20.2 of the Vienna Code retroactively permits the formation of names based on non-Latin terms, and, thereby, validates *Cleistogenes* and retroactively makes *Kengia* illegitimate and superfluous. Yu and Zhao (2005) have suggested that there are 18 species and eight varieties of *Kengia* in China. In Flora of China, there are 10 species and a variety, including *C. squarrosa*, *C. songorica*, *C. ramiflora*, *C. mucronate*, *C. festucacea*, *C. caespitosa* var. *caespitosa*, *C. kitagawae*, *C. polyphylla*, *C. hackelii* var. *hackelii*, *C. hancei*, and *C. hackelii* var. *nakaii* (Chen et al., 2006). Moreover, 13 records have been accepted in the plant list^1^, including *C. caespitosa* var. *caespitosa*, *C. festucacea*, *C. gatacrei*, *C. hackelii* var. *hackelii*, *C. hancei*, *C. kitagawae*, *C. mucronate*, *C. nedoluzhkoi*, *C. polyphylla*, *C. ramiflora*, *C. serotine*, *C. songorica*, and *C. squarrosa*. Two species including *C. songorica* and *C. polyphylla* have been shown as successive early diverging groups based on seven plastid regions and ITS regions, while the relationships among the remaining taxa remain largely unexplored (Peterson et al., 2016). Therefore, more evidence is required to clarify intrageneric relationships of *Cleistogenes*.

As an important organelle in plant cells, chloroplast participates in many biological processes (Leister, 2003; Lancien et al., 2006; Leister and Kleine, 2008). Chloroplast genome (plastome) of angiosperm usually has a typical quadripartite structure with two inverted repeat (IR) regions separated by a large single-copy (LSC) region and a small single-copy (SSC) region (Jansen and Ruhlman, 2012). Plastomes usually contain 110–130 genes, including ∼80 protein-coding genes (PCGs), ∼30 transfer RNA (tRNAs) genes, and four ribosomal RNA (rRNA) genes (Guisinger et al., 2010). Although plastomes are conserved in grass species, they exhibit different levels of sequence divergence in different regions of plastomes, such as IR regions, PCGs, and intergenic regions. Plastome sequences can offer valuable insights into phylogenetic relationships in plants (Dong et al., 2012; Curci et al., 2016). They have been increasingly adopted in phylogenetic studies of Poaceae species due to the rapid development of next-generation sequencing technology (Saarela and Graham, 2010; Burke et al., 2016; Duvall et al., 2016, 2020; Guo et al., 2019; Orton et al., 2019; Liu et al., 2020; Welker et al., 2020; Hardion et al., 2021). Plastomes have been used to study phylogenetic relationships within *Eragrostis* and *Triodia* of Chloridoideae (Anderson et al., 2019; Somaratne et al., 2019). Relationships among tribes and some genera of chloridoid grasses have been clarified based on the complete plastome (Duvall et al., 2016).

In the present work, we aimed to: (1) explore the plastomes of *Cleistogenes* and its closely related genera; (2) determine the phylogenetic placement of *Cleistogenes* within *Ael-Cle-Ori-Trio* clade (*Aeluropus*, *Cleistogenes*, *Orinus*, and *Triodia*); and (3) study the intrageneric relationships of *Cleistogenes*. A total of 15 plastomes were sequenced and assembled, including 12 *Cleistogenes* taxa (including 8 species and 4 varieties), two *Tripogon* species, and one *Aeluropus* species. Collectively, we, for the first time, comprehensively analyzed *Cleistogenes* plastomes.

## Materials and Methods

### Taxon Sampling, DNA Extraction, and Sequencing

In the present study, 32 plastomes representing 32 taxa were included in the phylogenetic analysis. Among them, 15 taxa including two outgroup taxa (*Tripogon bromoides* and *T. chinensis*) were newly sequenced, and the other 17 taxa were downloaded from GenBank [*Aeluropus lagopoides* (NC_042858), *Orinus kokonorica* (NC_042859), *Triodia basedowii* (NC_042860), *T. chichesterensis* (NC_042861), *T. concinna* (NC_042862), *T*. *glabra* (NC_042863), *T. lanigera* (NC_042872), *T. longiceps* (NC_042864), *T. mallota* (NC_042865), *T. plurinervata* (NC_042866), *T. rigidissima* (NC_042867), *T. schinzii* (NC_042870), *T. scintillans* (NC_042871), *T. stipoides* (NC_037157), *T. tomentosa* (NC_042868), *T. vanleeuwenii* (NC_042869), *T. wiseana* (NC_037161)]. Fresh leaves of these 15 taxa were collected in the field, followed by the drying process with silica gel. Voucher specimens and silica-dried leaves were stored at the College of Life Sciences, Shandong Normal University (SDNU), Jinan, China. Table 1 lists the sampling locality and GenBank accession numbers of these taxa. Total genome DNA was extracted using a modified CTAB method (Doyle and Doyle, 1987). The isolated DNA was subjected to agarose gel electrophoresis to assess the DNA quality. The concentration of total DNA was determined using a NanoDrop 2000 spectrophotometer (Thermo Scientific, Wilmington, DE, United States). A final DNA concentration of >30 ng/μL was selected for library preparation. Sequencing library was generated using NEB Next^®^ Ultra^TM^ DNA Library Prep Kit (Illumina, NEB, United States). Genomic DNA were fragmented by sonication to a size of 350 bp. Then DNA fragments were end-polished, A-tailed, and ligated with the full-length adapter for Illumina sequencing, followed by further PCR amplification. DNA libraries were sequenced through the Illumina NovaSeq 6000 platform and 150 bp paired-end reads were generated at Novogene (Beijing, China).

**TABLE 1:** The information of the sequenced taxa in the present study.

Taxon	Locality	GenBank accession number
*Aeluropus sinensis*	Luoyang, Henan, China	MW194080
*Cleistogenes caespitosa* var. *caespitosa*	Lingyuan, Liaoning, China	MW194082
*Cleistogenes caespitosa* var. *ramosa*	Qingdao, Shandong, China	MW194083
*Cleistogenes chinensis*	Qingdao, Shandong, China	MW194084
*Cleistogenes festucacea*	Jinan, Shandong, China	MW194085
*Cleistogenes gracilis*	Jinan, Shandong, China	MW194086
*Cleistogenes hackelii* var. *hackelii*	Jinan, Shandong, China	MW194087
*Cleistogenes hackelii* var. *nakaii*	Jinan, Shandong, China	MW194088
*Cleistogenes hancei*	Jinan, Shandong, China	MW194089
*Cleistogenes polyphylla*	Jinan, Shandong, China	MW194090
*Cleistogenes songorica*	Xi’an, Shaanxi, China	MW194091
*Cleistogenes* sp. nov.	Jinan, Shandong, China	MW194081
*Cleistogenes squarrosa*	Hohhot, Inner Mongolia, China	MW194092
*Tripogon bromoides*	Qingdao, Shandong, China	MW194093
*Tripogon chinensis*	Qingdao, Shandong, China	MW194094

### Plastome Assembly and Annotation

After sequencing data were obtained, plastomes were assembled as described in Qu et al. (2019a). Both the Organelle Genome Assembler (OGA) pipeline and Spades v3.13.0 (Bankevich et al., 2012) were used in plastome assembly. The latter one was with the “careful”-option and k-mers of 61, 81, 101, and 121. Annotation was carried out using PGA (Qu et al., 2019b). A manual correction was conducted using Geneious v9.1.4. Plastome sequences downloaded from the NCBI database were re-annotated using PGA, followed by manual correction. The circular maps for newly sequenced plastomes were generated using the OGDRAW tool (Stephan et al., 2019).

### Repeat Sequence Analysis

MISA was adopted to detect the simple sequence repeats (SSRs) of 32 taxa (Beier et al., 2017), and the thresholds of 8, 4, 4, 3, 3, and 3 repeat units were set for mono-, di-, tri-, tetra-, penta-, and hexa-nucleotides, respectively (Vieira et al., 2016). REPuter^2^ was used to identify palindromic, direct, reverse, and complement repeats, with a hamming distance set to 3, a minimum repeat size set to 30 bp, and a sequence identity set to >90% (Kurtz et al., 2001).

### Codon Usage Analysis

Referring to the unequal use of synonymous codons in an organism, codon usage can be used to determine the gene expression level, etc. Codon usage analysis of 32 taxa was performed on PCGs. The PCGs shorter than 300 bp were removed to avoid sampling bias (Wright, 1990; Rosenberg et al., 2003). The frequency of the nucleotides G + C at the third position (GC3s), the frequency of each base A, T, G, and C at the third position of codons (A3s, T3s, G3s, and C3s), the relative values of synonymous codon usage (RSCU) and GC content were determined using CodonW v1.4.2^3^. RSCU is a simple measure of non-uniform usage of synonymous codons in a coding sequence. If RSCU value of a codon >1, that codon is frequently used than expected whereas RSCU value < 1, means that the codon is less frequently used than expected. If RSCU equals 1, it means that the codon is used randomly and equally with other synonymous codons (Sharp et al., 1986).

### Sequence Divergence Analysis

Both the PCGs and non-coding regions (NCRs) longer than 200 bp were aligned using MAFFT v7.313 (Katoh and Standley, 2013). Variable sites and parsimony-informative (parsim-info) sites were calculated using Mega v7.0 (Sudhir et al., 2016). Whole plastome comparison of 12 *Cleistogenes* taxa (including 8 species and 4 varieties), two *Aeluropus* species, 15 *Triodia* species, two *Tripogon* species, and one *Orinus* species was performed by mVISTA (Frazer et al., 2004) using *A. lagopoides* as the reference.

### Phylogenetic Analysis and Divergence Time Estimations

One copy of IR was removed before the phylogenetic analysis. All 32 taxa were used for phylogenetic analyses of complete plastomes, PCGs, and NCRs. Sequences were aligned using MAFFT v7.313 (Katoh and Standley, 2013). The maximum likelihood (ML) tree was constructed using RAxML v8.2.10 (Alexandros, 2014) with 1,000 bootstrap replicates and the GTR + I + G model. The best model of evolution was determined using jModelTest2 (Darriba et al., 2012). The Bayesian inference (BI) tree was constructed using MrBayes v3.2.6 with The Markov Chain Monte Carlo (MCMC) was run for 1,000,000 steps with a random starting tree, birth–death default priors, and sampled one tree every 1,000 steps. We discarded the first 25% steps as burn-in. A relaxed clock method and penalized likelihood were involved in dating analyses using treePL (Sanderson, 2002; Smith and O’Meara, 2012). We generated 1,000 ML bootstrap trees with branch lengths by using RAxML. The minimum age of the *Tripogon* crown node was constrained at 20 Ma (Anderson et al., 2019). The maximum age of *Aeluropus*, *Cleistogenes*, *Orinus*, *Triodia*, and *Tripogon* crown node was assigned as 25 million years ago (Ma) (Anderson et al., 2019). The minimum age of *Aeluropus*, *Cleistogenes*, *Orinus*, *Triodia*, and *Tripogon* crown was assigned as 7.9 Ma (Anderson et al., 2019). The minimum and maximum age for the internal nodes were calculated from dating 1,000 bootstrap trees by using treePL and TreeAnnotator v1.8.495 (Drummond et al., 2012).

## Results

### Characteristics of *Ael-Cle-Ori-Trio* Clade Plastomes

**FIGURE 1:**
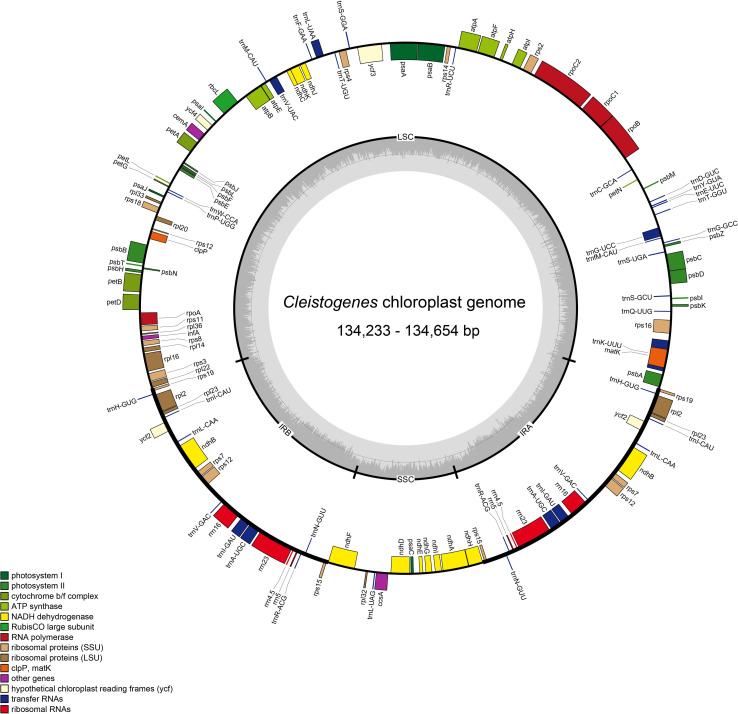
Plastome map of twelve *Cleistogenes* taxa. Genes on the inside of the map are transcribed in the clockwise direction, while genes on the outside of the map are transcribed in the counterclockwise direction. The dashed darker gray area in the inner circle indicates the GC content, while the lighter gray area shows the AT content. IR, inverted repeat; SSC, small single-copy; LSC, large single-copy.

The size of the 12 *Cleistogenes* plastomes ranged from 134,233 bp (*C. caespitosa* var. *ramosa*) to 134,654 bp (*C. caespitosa* var. *caespitosa*). All of them had a typical circular quadripartite structure as most angiosperms, consisting of an LSC region (80,003–80,430 bp), an SSC region (12,648–12,671 bp), and a pair of IR regions (20,780–20,782 bp) (Figure 1 and Table 2). The GC content of these plastomes was 38.4%. The values of total genes, total PCGs, total tRNA, total rRNA, unique genes, and unique PCGs are the same for all taxa analyzed. A total of 132 genes (111 unique) were annotated, including 86 PCGs (77 unique), 38 tRNA genes (30 unique), and eight rRNA genes (four unique). These above-mentioned genes were in the same order and could be assigned into four groups as follows: photosynthesis-related genes, self-replication-related genes, other genes, and functionally unknown genes (Supplementary Table 1). Moreover, 10 PCGs had introns (*atpF*, *ndhA, ndhB, petB*, *petD*, *rpl2*, *rpl16, rps12*, *rps16*, and *ycf3*), two (*rps12* and *ycf3*) of them had two introns, and the other eight genes had a single intron. The *rps12* was a *trans-*spliced gene, with the 5′-end exon located in the LSC region, while its 3′-end exon and intron were duplicated and located in the IR regions. Seven PCGs (*rps19*, *rpl2*, *rpl23*, *ycf2*, *ndhB*, *rps7*, and *rps15*), eight tRNA genes (*trnH-GUG*, *trnI-CAU*, *trnL-CAA*, *trnV-GAC*, *trnI-GAU*, *trnA-UGC*, *trnR-ACG*, and *trnN-GUU*) and four rRNA genes (*rrn16*, *rrn23*, *rrn4.5*, and *rrn5*) were duplicated in the IR regions.

**TABLE 2:** Summary of major characteristics of the 32 plastomes.

Taxon	Plastome length (bp)	LSC length (bp)	IR length (bp)	SSC length (bp)	GC content (%)
*Aeluropus lagopoides*	135,518	80,813	21,010	12,685	38.2
*Aeluropus sinensis*	135,563	80,855	21,010	12,688	38.3
*Cleistogenes caespitosa* var. *caespitosa*	134,654	80,430	20,780	12,664	38.4
*Cleistogenes caespitosa* var. *ramosa*	134,233	80,003	20,780	12,670	38.4
*Cleistogenes chinensis*	134,550	80,337	20,780	12,653	38.4
*Cleistogenes festucacea*	134,594	80,375	20,780	12,659	38.4
*Cleistogenes gracilis*	134,576	80,357	20,780	12,659	38.4
*Cleistogenes hackelii* var. *hackelii*	134,588	80,369	20,780	12,659	38.4
*Cleistogenes hackelii* var. *nakaii*	134,531	80,318	20,780	12,653	38.4
*Cleistogenes hancei*	134,549	80,329	20,781	12,658	38.4
*Cleistogenes polyphylla*	134,579	80,360	20,780	12,659	38.4
*Cleistogenes songorica*	134,556	80,348	20,780	12,648	38.4
*Cleistogenes* sp. nov.	134,497	80,266	20,780	12,671	38.4
*Cleistogenes squarrosa*	134,509	80,296	20,782	12,649	38.4
*Orinus kokonorica*	133,953	80,650	20,327	12,649	38.2
*Triodia basedowii*	135,278	80,563	21,037	12,641	38.4
*Triodia chichesterensis*	135,284	80,625	21,012	12,635	38.4
*Triodia concinna*	135,218	80,562	21,009	12,638	38.5
*Triodia glabra*	135,251	80,576	21,019	12,637	38.4
*Triodia lanigera*	135,453	80,774	21,019	12,641	38.4
*Triodia longiceps*	134,425	80,341	20,708	12,668	38.4
*Triodia mallota*	135,261	80,575	21,024	12,638	38.4
*Triodia plurinervata*	135,386	80,880	21,027	12,452	38.5
*Triodia rigidissima*	135,657	80,989	21,023	12,622	38.4
*Triodia schinzii*	135,016	80,950	20,712	12,642	38.3
*Triodia scintillans*	135,301	80,592	21,037	12,635	38.4
*Triodia stipoides*	134,874	80,169	21,047	12,611	38.4
*Triodia tomentosa*	135,375	80,703	21,024	12,624	38.5
*Triodia vanleeuwenii*	135,318	80,656	21,013	12,636	38.4
*Triodia wiseana*	134,962	80,898	20,711	12,642	38.4
*Tripogon bromoides*	133,744	79,120	21,013	12,598	38.5
*Tripogon chinensis*	133,744	79,121	21,013	12,597	38.5

*LSC, large single-copy region; IR, inverted repeat region; SSC, small single-copy region.*

Among all the 32 taxa, the plastomes of two *Tripogon* taxa (133,744 bp) were the smallest, while the plastome of *Triodia rigidissima* (135,657 bp) was the largest (Table 2). The length of LSC and SSC ranged from 80,003 bp (*C. caespitosa* var. *ramosa*) to 80,989 bp (*T. rigidissima*), and from 12,452 bp (*T. plurinervata*) to 12,688 bp (*A. sinensis*), respectively. The GC content ranged from 38.2 to 38.5%. All these plastomes encoded 132 genes (111 unique), including 86 PCGs (77 unique), 38 tRNA genes (30 unique), and eight rRNA genes (four unique).

### Repeat Sequence Analysis

A total of 1,687 repeats were identified in these 32 plastomes by Reputer, including 1,012 forward, 650 palindromic, 24 reverse, and one complement. The sole complement repeat was only identified in *C. chinensis*. The number of repeats ranged from 45 (*T. stipoides*) to 76 (*T. lanigera*). Most taxa had 45–60 pairs of repeats. The repeat lengths in 30–39 bp were most common, accounting for 64.1%. Repeats with the lowest proportion (7.1%) ranged in size from 50 to 59 bp. Repeat lengths in 50–59 bp were not identified in *Cleistogenes* taxa.

**FIGURE 2:**
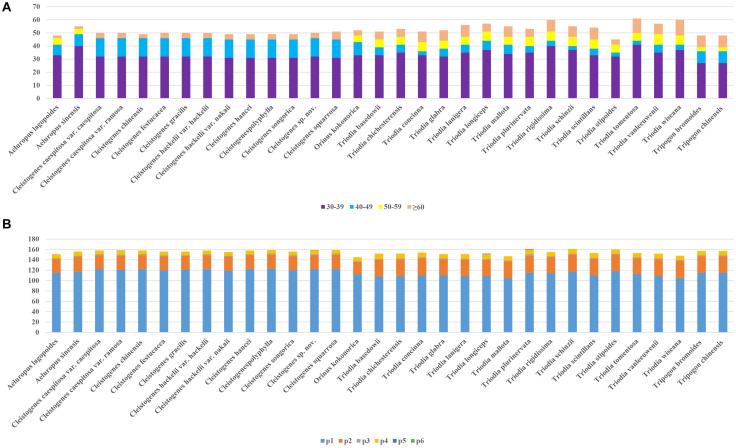
Repeats in 32 plastomes. **(A)** The number of repetitive sequences by lengths; **(B)** The number of different types of SSRs (p1–p6 indicate mono-, di-, tri-, tetra-, penta-, hexanucleotides, respectively).

Figure 2 shows that the number of SSRs for all taxa ranged from 145 (*Orinus kokonorica*) to 161 (*T. plurinervata* and *T. schinzii*). The number of SSRs found in *Cleistogenes* taxa ranged from 155 to 159. There were six categories of SSRs detected in these taxa, including mono-nucleotide, di-nucleotide, tri-nucleotide, tetra-nucleotide, penta-nucleotide, and hexa-nucleotide repeats. In all taxa, mono-nucleotides were the most common SSRs, accounting for 74.4%, followed by di-nucleotide (19.1%). There were some types of SSRs found in all plastomes, including mono-(A/C/G/T), di-(AG/AT/CT/GA/TA/TC), tri-(AAT), and tetra-(AACG/AATA/GTAG/YCGT/TTCT) (Supplementary Table 2). Penta-nucleotide repeats were only found in *T. longiceps* and *T. plurinervata*, and hexa-nucleotide repeats (AAATAT) were only found in *Cleistogenes* sp. nov. and *C. caespitosa* var. *ramosa*.

### Codon Usage Analysis

A total of 48 PCGs were used in the codon usage analysis. The number of codons identified in the codon usage analysis ranged from 16,633 (*T. stipoides*) to 16,678 (*T. tomentosa*) (Supplementary Table 3). The GC contents of the entire plastomes, the frequency of the nucleotides G + C at the third position (GC3s), and the frequency of each base A, T, G, and C at the third position of codons (A3s, T3s, G3s, and C3s) were similar in all taxa. The GC content was approximately 39%, suggesting an abundance of AT. A3s and T3s were larger than G3s and C3s. Leucine was the most frequent amino acid in the plastomes (∼10.8%), while cysteine was the least frequent amino acid (∼1.0%) (Supplementary Table 4). The relative values of synonymous codon usage (RSCU) were determined to estimate the preference for the use of synonymous codons. In all taxa, nearly all the amino acid codons had a bias (RSCU > 1 or RSCU < 1) except for methionine (AUG, RSCU = 1) and tryptophan (UGG, RSCU = 1).

### IR Expansion and Contraction of *Ael-Cle-Ori-Trio* Clade Plastomes

**FIGURE 3:**
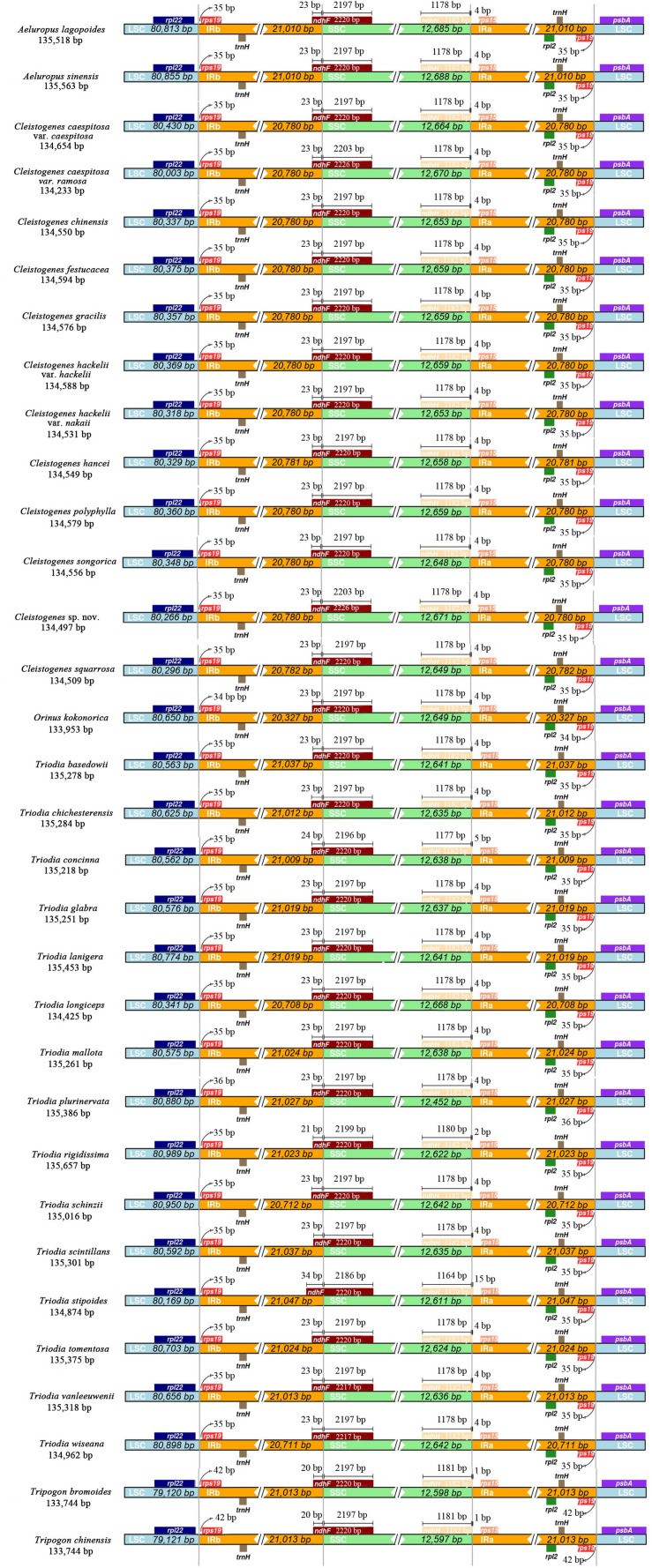
Comparison of the boundaries between LSC/SSC and IR regions among the 32 plastomes.

The IR/SSC and IR/LSC boundary regions of these taxa were very conserved (Figure 3). The junctions of LSC/IRb and IRa/LSC were located in intergenic regions. LSC/IRb was located between *rpl22* and *rps19*. The fragment size of *rpl22-rps19* located in the IRb region was 34 bp in *O. kokonorica*, 36 bp in *T. plurinervata*, 42 bp in two *Tripogon* species, and 35 bp in other taxa. IRa/LSC was located between *rps19* and *psbA*. The length of *rps19-psbA* located in the IRa region was 35 bp in most taxa, 34 bp in *O. kokonorica*, 36 bp in *T. plurinervata*, and 42 bp in two *Tripogon* species. The IRb/SSC boundaries were located in the *ndhF* gene, and part of this gene was duplicated from 20 to 34 bp in the IRb region. The *ndhH* gene crossed the SSC/IRa region in all taxa, and this gene extended 1 to 15 bp into the IRa region.

### Sequence Divergence Analysis

**FIGURE 4:**
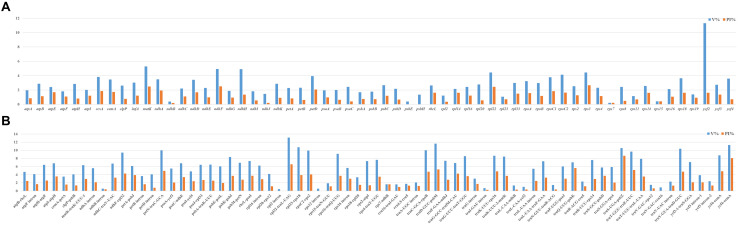
Comparison of percentage of parsim-info sites among 32 plastomes. **(A)** PCGs, *X*-axis: name of PCGs, *Y*-axis: percentage of variable sites; **(B)** NCRs, *X*-axis: name of NCRs, *Y*-axis: percentage of parsim-info sites.

**FIGURE 5:**
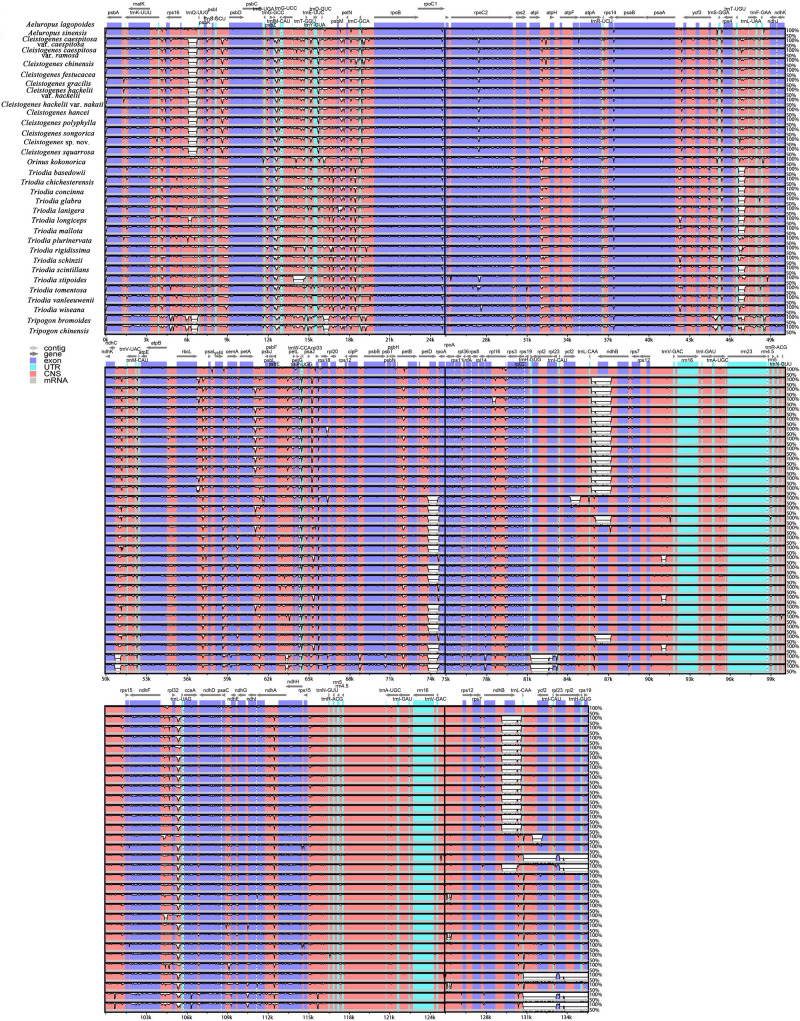
Comparative analysis of the plastomes of 32 taxa. The plastome of *Aeluropus lagopoides* was used as a reference. The vertical scale indicates the percentage of identity, ranging from 50 to 100%. Genome regions are color-coded as exon, UTR, and conserved non-coding sequences (CNS).

To compare the sequence divergence among all the taxa, variable and parsim-info sites were calculated for PCGs and NCRs (Figure 4). The sequence divergence of NCRs was generally higher compared with the PCGs. For the PCGs, the percentage of parsim-info (Pi%) sites ranged from 0% (*psbE* and *psbH*) to 4.44% (*rps3*), and the percentage of variable sites ranged from 0.21% (*rps7*) to 11.30% (*ycf2*). For the NCRs, the percentage of parsim-info sites ranged from 0% (*rpl2 intron*, *rps12 intron* and *trnV-GAC-rrn16*) to 12.48% (*trnS-UGA-psbZ*), and the percentage of variable sites ranged from 0.45% (*rpl2 intron*) to 13.12% (*rpl32-trnL-UAG*). Five PCGs with a high percentage of parsim-info sites were selected, including *rps3*, *ndhF*, *matK*, *rpl22* and *petD*. Five NCRs were identified, including *trnS-UGA-psbZ*, *rpl32-trnL-UAG*, *trnQ-UUG-psbK*, *trnD-GUC-psbM*, *trnT-GGU-trnE-UUC*. The mVISTA results showed that all aligned plastome sequences were highly similar, and the NCRs showed a higher divergence compared with the PCGs (Figure 5).

### Phylogenetic Analysis and Divergence Time Estimations

**FIGURE 6:**
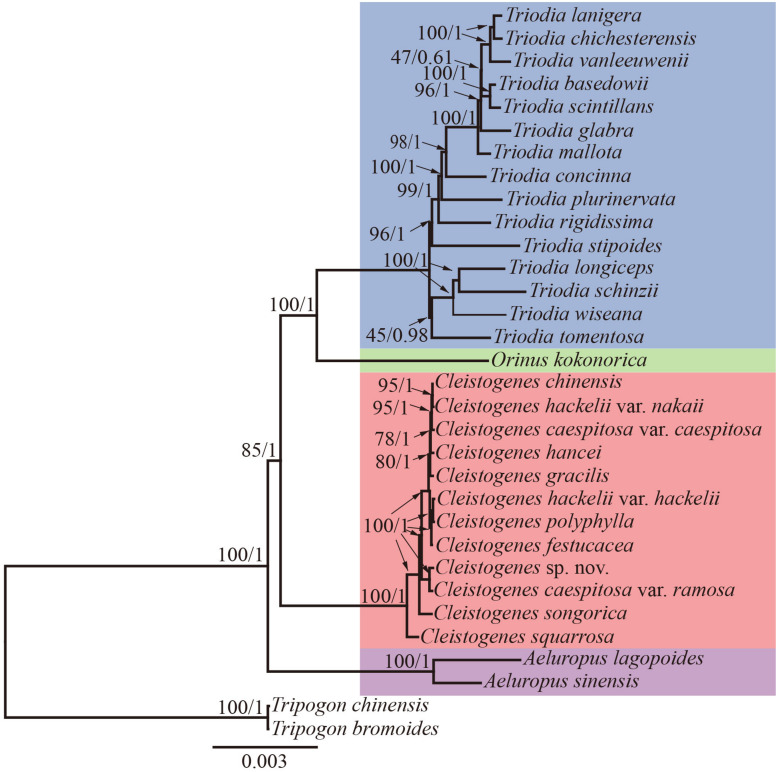
The ML phylogeny of *Cleistogenes* and its closely related genera based on complete plastomes. The obtained bootstrap values (BS) and Bayesian inference posterior probabilities (PP) are marked above the tree node (BS/PP). *Cleistogenes* taxa (including 8 species and 4 varieties) are highlighted in red, *Orinus* species are highlighted in green, *Triodia* species are highlighted in blue, and *Aeluropus* species are highlighted in purple.

**FIGURE 7:**
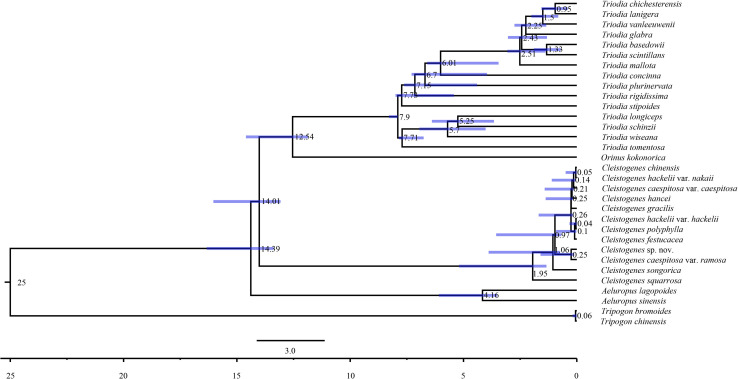
Chronogram of 32 taxa based on complete plastomes inferred by using treePL. Blue bars represent the minimum and maximum estimation of node ages.

Maximum likelihood and Bayesian inference phylogenetic analyses using three datasets (complete plastomes, PCGs, and NCRs) generated identical topologies for *Cleistogenes* and its closely related genera in the present study (Figure 6, Supplementary Figures 1–6). *Aeluropus lagopoides* and *A. sinensis* form a monophyletic clade with a strong support (BS = 100, PP = 1.00). *Cleistogenes* is strongly supported as a monophyletic group in ML and BI analysis (BS = 100, PP = 1.00). The clade composed of *Orinus* and *Triodia* (BS = 100, PP = 1.00) is sister to *Cleistogenes*. The sister relationship of *Cleistogenes* and *Orinus*-*Triodia* clade have a BS value of 85 and a PP value of 1. *Cleistogenes* taxa are grouped into four clades. Two species including *C. squarrosa* and *C. songorica* are the successive early diverging groups (BS = 100, PP = 1.00). The other 10 taxa are strongly supported to be divided into two groups: *Cleistogenes* sp. nov. and *C. caespitosa* var. *ramosa* form a monophyletic group (BS = 100, PP = 1.00) sister to other eight *Cleistogenes* taxa, and the clade composed of eight taxa (*C. hackelii* var. *hackelii*, *C. polyphylla*, *C. festucacea*, *C. hancei*, *C. hackelii* var. *nakaii*, *C. chinensis*, *C. caespitosa* var. *caespitosa*, and *C. gracilis*) are detected with short internal branch length. The results of divergence time estimations were shown in Figure 7 and Supplementary Table 5. The divergence time between *Cleistogenes* and *Orinus*-*Triodia* was estimated at 14.01 Ma (13.07 to 16.04 Ma) in Miocene. The split between *Triodia* and *Orinus* was estimated at 12.54 Ma (11.86 to 14.6 Ma) in Miocene. The result indicated that *Cleistogenes* began to diversify in Pliocene or Pleistocene (1.34–5.2 Ma).

### Morphological Comparison of *O. kokonorica* and *C. songorica*

**FIGURE 8:**
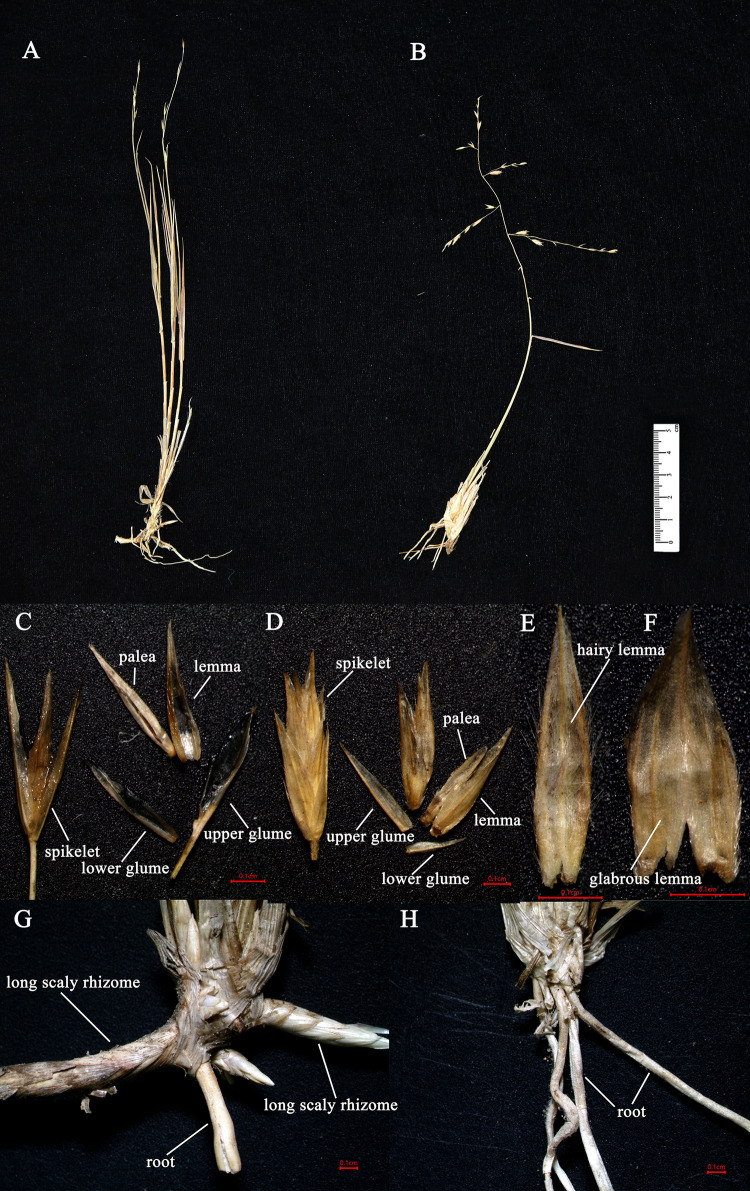
The morphology of *O. kokonorica* and *C. songorica*: **(A)** individual of *O. kokonorica*; **(B)** individual of *C. songorica*; **(C)** spikelet of *O. kokonorica*; **(D)** spikelet of *C. songorica*; **(E)** lemma of *O. kokonorica*; **(F)** lemma of *C. songorica*; **(G)** underground part of *O. kokonorica*; **(H)** underground part of *C. songorica*.

Spikelet, lemma and underground part of *O. kokonorica* and *C. songorica* were compared in the present study (Figure 8). Spikelets of *O. kokonorica* and *C. songorica* were laterally compressed, fewer florets in *O. kokonorica. O. kokonorica* is charactered by lemma thin, dorsally black-brown but yellow-brown at base and apex, with loosely pilose at margins and lower keel. Lemma of *C. songorica* was wider than *O. kokonorica* and glabrous. Underground part of these two taxa were significant different. Long scaly rhizomes can be observed in this part of *O. kokonorica*, while they were not present in *C. songorica*.

## Discussion

### Plastome Evolution

Plastomes of land plants have greatly conserved genome size, gene content, gene order, and organization (Palmer, 1991; Bock, 2007). Previous studies have assessed the organization and evolution of Poaceae plastomes (Katayama and Ogihara, 1993; Guisinger et al., 2010). In the present study, the plastomes had high structural similarity among all taxa, including genome size, gene content, and gene order. The plastomes of these taxa had an average genome size of approximately 135 kb. Plastomes of these taxa showed the typical quadripartite structure as previously reported Poaceae species, consisting of an LSC region and an SSC region, and separated by a pair of IR regions (Rousseau-Gueutin et al., 2015; Huang et al., 2017). Each plastome contained the same number of genes (86 PCGs, 38 tRNAs, and eight rRNAs), with a similar GC content and conserved intron positions. There were some unusual features of Poaceae plastomes compared with other angiosperm species, including three gene loss (*accD*, *ycf1*, and *ycf2*) and intron loss from two genes (*clpP* and *rpoC1*) (Katayama and Ogihara, 1996; Guisinger et al., 2010). In the present study, *accD* and *ycf1* were not annotated in all taxa. These two genes might be useful molecular markers for phylogenetic analysis of land plants, playing essential roles in leaf development (Kode et al., 2005) and plant viability (Kikuchi et al., 2013). In the case of *ycf2* gene loss, different lengths of *ycf2* were found, suggesting a progressive degradation. The *ycf2* gene retained segments in different lengths, ranging from 105 to 792 bp. The *ycf2* gene plays a significant undetermined function in higher plants (Drescher et al., 2000). This gene can provide effective variable sites for reconstructing a generally well-supported phylogeny (Huang et al., 2010). Intron losses of *clpP* and *rpoC1* were detected in all 32 taxa. Gene and intron losses can lead to a decrease in grass plastome size (Zhang J. et al., 2011).

The expansion and contraction of the IR region have been proposed as an important source of length variation in plastomes (Palmer et al., 1987; Aii et al., 1997; Liu et al., 2018). However, in our present study, all taxa exhibited a highly conserved pattern of IR boundaries with only slight structural variations. In Poaceae, *ndhH* and *ndhF* are located near opposite ends of the SSC region and can extend into IR regions (Maier et al., 1990; Davis and Soreng, 2010). In the early diverged grasses and closest relatives, *ndhH* extends 175 to 200 nucleotides into the IR region, while *ndhF* is confined to the SSC region (Davis and Soreng, 2010). Davis and Soreng (2010) have reported that a part of *ndhH* and *ndhF* less than 30 nucleotides migrates into IR regions in PACMAD clade of the Poaceae family. A similar pattern has been observed in Eragrostideae species except for *Eragrostis tenellula* (Somaratne et al., 2019). In our present study, this phenomenon was observed in most taxa, except for *Triodia stipoides*, in which *ndhF* extended 34 nucleotides into the IRb region. The different migrations of gene termini relative to the two SSC-IR junctions are also of significance in terms of the gene overlap (Davis and Soreng, 2010).

As tandemly repetitive DNA sequences, SSRs show high levels of polymorphism. They are abundant across plastomes. As they are reliable and highly polymorphic, they have been increasingly used in phylogenetics, species identification and population genetic studies (Kalia et al., 2011). Similar to the results of other Poaceae species, the predominant type of SSRs is mono-nucleotides, of which A or T repeats account for the majority (Tanaka et al., 2016; Somaratne et al., 2019). The abundance and distribution pattern of penta-nucleotide and hexa-nucleotide repeats were different in the present study. Penta-nucleotide repeats and hexa-nucleotide repeats are the most common types among all the *Oryza* species (Tripathy et al., 2019), while they were species-specific in the present study. Penta-nucleotide repeats were only found in *T. longiceps* and *T. plurinervata*, and hexa-nucleotide repeats (AAATAT) were only detected in *Cleistogenes* sp. nov. and *C. caespitosa* var. *ramosa*. In the present study, penta-nucleotide repeats were distributed in both coding regions and NCRs, while hexa-nucleotide repeats were distributed in NCRs. Repeats identified by Reputer were assigned into four categories, including forward, reverse, complement, and palindromic. Most of the repeats were forward and reverse repeats. All the identified repeats in the present study might be useful in future studies on population genetics of these 32 taxa.

Synonymous codon usage bias represents the differences in the relative frequency of synonymous codons for individual amino acids in protein-coding sequences, and many biological factors, such as gene expression, amino acid composition of protein, and protein structure, are correlated to such bias (Angellotti et al., 2007). Base composition is a balance between G + C nucleotide pairs and mutational pressure, which is a pervasive effect on codon usage (Sueoka, 1962). In the present study, the GC content was highly conserved, suggesting that these plastomes achieved evolutionary equilibrium (Zhang Y. R. et al., 2012). Our findings were consistent with previously reported Poaceae plastomes that all the 32 plastomes had similar codon usage patterns, and the AT-rich bias was the strongest in the third codon position (Somaratne et al., 2019). Preferred codons could be useful in designing degenerate primers, the introduction of point mutation, and evolutionary studies. In the standard genetic codes, there are 64 codons, 61 of which encode 20 different amino acids, and 3 of which are stop codons. All amino acids are encoded by two to six synonymous codons except for Met and Trp. Among the stop codons (UAA, UAG, and UGA), the predominantly used one was UAA (RSCU > 1.63) in the present study. Similar favorite stop codons have been found in some Poaceae species, e.g., *Zizania latifolia* (Zhang et al., 2016) and *Pennisetum glaucum* (Raveendar et al., 2019).

### Phylogenetically Informative Markers

Plastid regions are widely used in the phylogenetic study of Poaceae (Johnson, 1997; Bouchenak-Khelladi et al., 2008; Rua et al., 2010; Barbera et al., 2020). Previous studies of *Cleistogenes* and its closely related genera mainly include 11 plastid regions (*ndhA*, *ndhA intron*, *ndhF*, *rps16-trnK*, *rps16 intron*, *rps3*, *rpl32-trnL*, *rps16*, *rpoC2*, *ccsA*, and *matK*), while these regions are not effective in resolving the intra- and intergeneric relationships of *Cleistogenes*. Some of them (e.g., *rps16*, *rpoC2*, *ndhA* intron, and *rps16* intron) had a low percentage of parsim-info sites (Figure 4). Therefore, phylogenetic trees based on these regions generated low resolution and different topologies. In the present study, *rps3* was the most divergent PCG, while Pi% was only 4.44%. Insufficient sequence polymorphism of the markers hindered their use for phylogenetic study. Therefore, it is important to identify more informative markers to study the intra- and inter-genus phylogeny for *Cleistogenes*.

In Poaceae plastomes, PCGs and IR regions are more conserved at the sequence divergence level compared with NCRs and single-copy (SC) regions, respectively (Bortiri et al., 2008; Qiu et al., 2019; Somaratne et al., 2019). In the present study, the whole plastome comparative analysis showed that IR regions were more conserved compared with the SC regions, and comparison of the percentage of parsim-info sites confirmed that NCRs had higher Pi% than PCGs. NCRs evolve via accumulating nucleotide substitutions and microstructural mutations (insertions, deletions, and inversions) (Yamane et al., 2006). Currently, NCRs of plastomes have become an important tool in studying phylogenetic relationships in Poaceae (Liu et al., 2008; Zhang Y. X. et al., 2012; Oliveira et al., 2014). In the present study, five regions displayed remarkably higher Pi% (>5%), including four regions (*trnS-UGA-psbZ*, *trnQ-UUG-psbK*, *trnD-GUC-psbM*, *trnT-GGU-trnE-UUC)* located in the LSC region and one region (*rpl32-trnL-UAG*) located in the SSC region. These regions might be potential molecular markers in the phylogenetic analysis of *Cleistogenes* and its related genera. Among the five phylogenetically informative markers, two regions (*rpl32-trnL-UAG* and *trnT-trnE*) have been reported as highly variable markers to study phylogenetic relationships of Poaceae species (Rousseau-Gueutin et al., 2015; Qiu et al., 2019).

### Whether Orininae Should Be Established?

The monophyly of the *Ael-Cle-Ori-Trio* clade has been strongly supported in previous studies (Soreng et al., 2015, 2017). However, there are some incongruences about the phylogenetic relationships among these four genera (Chen et al., 2006; Shan et al., 2010; Soreng et al., 2015, 2017; Peterson et al., 2016). In the present study, the analysis of complete plastomes strongly supported the highly congruent phylogenetic topology for *Ael-Cle-Ori-Trio* clade (Figure 6). *Aeluropus* species including *A. lagopoides* and *A. sinensis* form a monophyletic clade, which is located at the basal position in the tree. The *Cle*-*Ori*-*Trio* clade is detected with a high bootstrap value. *Cleistogenes* is strongly supported as a monophyletic group and sister to the clade composed of *Orinus* and *Triodia*. The position of *Cleistogenes* recovered here is incongruent with other studies. The placement of *Cleistogenes* is not resolved in the plastid tree based on seven plastid regions. However, *Cleistogenes* is sister to *Orinus* in the ITS tree (Peterson et al., 2010) and combined tree (Peterson et al., 2010, 2016). Subtribe Orininae has been newly proposed in a phylogenetic analysis of 213 species based on seven plastid regions and ITS (Peterson et al., 2016). The present study showed that *Cleistogenes* is sister to the clade composed of *Orinus* and *Triodia*, which indeed did not support the establishment of Orininae (Peterson et al., 2016). These three genera are different in morphology. Inflorescences of *Cleistogenes* and *Orinus* are sparse panicle (Chen et al., 2006). Inflorescence of *Triodia* is usually a panicle of solitary spikelets, and sometimes it is a spike or raceme (Lazarides, 1997). *Cleistogenes* is a genus of Eurasian flowering plants in the grass family. It is remarkable for cleistogamous spikelets hidden in the upper leaf sheaths (Chen et al., 2006), while cleistogamous spikelets are not found in *Orinus* and *Triodia*. Cleistogamy flowering assures plant reproduction under variable environmental conditions, and its development is known to be affected by drought, chilling, salinity, and light (Morinaga et al., 2008). *Orinus* is a genus of Asian plants in the grass family (Chen et al., 2006). *Triodia* is a large genus of hummock-forming grass endemic to Australia (Lazarides, 1997; Gamage et al., 2012). Eurasia and Australia have been separated by oceans at ca. 90 Ma (Davis et al., 2002), earlier than the origin of *Cle*-*Ori*-*Trio* clade. Therefore, long-distance dispersal must have played a dominant role in the transoceanic distribution of this clade. The split between these three genera go back to 14.01 Ma (Miocene). The Malay Archipelago probably facilitated biotic dispersal between Asia and Australia during the Miocene (Welzen et al., 2005). The habitat conditions of *Orinus* and *Triodia* were similar. They all distribute in arid regions on sandy or stony soils. Morphologically, leaf blades of *Orinus* and *Triodia* species are linear to involute and rigidly straight. Leaf involution is often hypothesized to confer survival value in xeric habitats by reducing stomatal and cuticular conductance and postponing desiccation. We compared the morphology of *O. kokonorica* and *C. songorica* (Figure 8). Lemmas of *Cleistogenes* are almost glabrous. Lemmas of *Orinus* are hairy all over or only on margins. Lemmas of *Triodia* species are entire or minutely to deeply 2- or 3-lobed and hairy. Trichomes (also called leaf hairs) are specialized cell types in the epidermal layer, that enhance the protection of plant tissues from the external factors both mechanically and chemically (Hauser, 2014). Long scaly rhizomes are recognized as an important character of *Orinus* (Chen, 1990; Chen et al., 2006). The scaly rhizomes allow the plants to survive unfavorable dry spells or seasons in dormancy and to re-sprout from the axils of the scale-like leaves when conditions improved. *Orinus*-*Triodia* and *Cleistogenes* may represent two different ways to adapt to extreme environments. Meanwhile, there are some similar micromorphological characters recognized in *Orinus* and *Triodia*. Papillae occurring on long cells and dumbbell-shaped silica cells are observed as two similar lemma micromorphological characters in *Triodia* and *Orinus*, while papillae and silica cells are absent in *Cleistogenes* taxa (Liu et al., 2010). From the above-mentioned results, some conflicts existed between plastome- and ITS-based trees. These conflicts could mostly result from rapid divergence, hybridization and incomplete lineage sorting. The *Cle*-*Ori*-*Trio* clade was estimated to have experienced rapid divergence in a short period (12.54 to 14.01 Ma), which could be an obstacle in resolving phylogenetic relationships within the clade. Natural hybridization and incomplete lineage sorting are very common in numerous plants (Ellstrand et al., 1996; Maddison, 1997). Hybridization would lead to alleles with mixed histories, therefore resulting in high character conflict (Folk et al., 2016). Incomplete lineage sorting may be defined as the failure for two or more allelic lineages to coalesce within a population (Degnan and Rosenberg, 2009). Hybridization and incomplete lineage sorting can lead to discord in phylogenetic analyses (Kubatko and Degnan, 2007). In conclusion, the establishment of Orininae was not supported by plastomes data.

### Phylogenetic Relationship Within *Cleistogenes*

*Cleistogenes* is a monophyletic genus in the tree based on complete plastomes, which reinforce the previous study using ITS and plastid sequences (Peterson et al., 2016). *Cleistogenes* began to diversify in Pliocene or Pleistocene (1.34–5.2 Ma). It is suggested that there is a climate shift in the Pliocene across large area of East Asia, as well as an even colder and drier Quaternary compared with the late Pliocene (Wang et al., 2019). As a kind of cool- and dry-adapted grass, *Cleistogenes* was probably diversified caused by climate change. The phylogenetic relationship among *Cleistogenes* taxa have been rarely studied. The lack of a well-supported intra-generic phylogenetic framework has posed a problem for the classification of *Cleistogenes*. Phylogenetic relationships within *Cleistogenes* recovered here are not congruent with the previous study. It is shown that *C. songorica* and *C. polyphylla* are early diverging groups in the phylogenetic analysis of Cynodonteae based on seven plastid regions and ITS regions (Peterson et al., 2016). In the present study, *C. songorica* and *C. squarrosa* are resolved as a highly supported successive sister group to the remaining 10 taxa. It is reported that *C. squarrosa* is a dominant grass species in grasslands (Wang et al., 2000; Liang et al., 2002). Dense foliage is on the low mounds of the culm, and upper internodes of the dried culm are curling and elongate. Awned spikelets are always with few flowers. These above-mentioned characteristics distinguish it from the remaining taxa of *Cleistogenes* (Chen et al., 2006). *C. songorica* is a dominant species of desert grassland, and it has excellent drought tolerance (Zhang J. et al., 2011; Zhang et al., 2014; Muvunyi et al., 2018). This species has dense tufts of gray-green leaves. Spikelets are purple and awnless, usually with broader lemmas. These two species including *C. songorica* and *C. squarrosa* can adapt to dry environment. In the complete plastome phylogenetic tree, *C. caespitosa* var. *ramosa* was sister to *Cleistogenes* sp. nov., while *C. caespitosa* var. *caespitosa* clusters with the other seven *Cleistogenes* taxa. The difference between this variety and its original variety was that the upper stem was much branched, and the panicle was extremely narrow. Similar morphological characters of *C. caespitosa* var. *ramose* and *C. caespitosa* var. *caespitosa* could be attributed to parallel evolution. Palea of *Cleistogenes* sp. nov. significantly shorter compared with the other *Cleistogenes* taxa. The clade composed of *C. hackelii* var. *hackelii*, *C. polyphylla*, *C. festucacea*, *C. hancei*, *C. hackelii* var. *nakaii*, *C. chinensis*, *C. caespitosa* var. *caespitosa*, and *C. gracilis* is found with short internal branch length. The short branches most likely represent a case of rapid radiation with lineage-specific heterogeneity in rates of substitution. *Cleistogenes* was estimated to have experienced rapid divergence within a short period, which could be a main reason for short internal branches between these 10 *Cleistogenes* taxa in phylogenetic tree. Lemma micromorphological characters of *C. caespitosa* var. *caespitosa*, *C. chinensis*, and *C. hackelii* var. *hackelii* are highly conserved (Liu et al., 2010). Straight outline of intercostal long cells, semi-circle-shaped cork cell, triangular-shaped stomata subsidiary cell, and papillate-based macro hair have been observed in *C. caespitosa* var. *caespitosa*, *C. chinensis*, and *C. hackelii* var. *hackelii*. In the present study. the phylogeny of complete plastomes revealed the intra-generic framework of *Cleistogenes*. However, future studies with a larger sample size at the population level or the next-generation sequencing approach may help resolve their phylogenetic relationships.

## Conclusion

Collectively, we sequenced, assembled, and annotated 15 plastomes of *Cleistogenes* and its closely related genera. Plastomes of 12 *Cleistogenes* taxa (including 8 species and 4 varieties), 15 *Triodia* species, one *Orinus* species, two *Tripogon* species, and two *Aeluropus* species were included in the present study. All the plastomes were highly conserved in plastome structure, gene content, gene order, and IR boundaries. The type and number of repeat sequences and SSRs were calculated and could be used in studies of phylogenetics, species identification, and population genetics. By examining variable sites and parsim-info sites, five highly variable regions were identified, which could be used for phylogenetics, population genetics, and biogeography of *Cleistogenes* and its closely related genera. Phylogenetic analysis revealed that *Cleistogenes* is sister to a clade composed of *Orinus* and *Triodia*, whereas recently proposed Orininae (*Cleistogenes* and *Orinus*) is not supported in the present study. It is suggested that *C. squarrosa* and *C. songorica* are the successive early diverging groups. The other 10 taxa are divided into two groups. *Cleistogenes* sp. nov. and *C. caespitosa* var. *ramosa* form a monophyletic group sister to the other eight *Cleistogenes* taxa. Our current findings provided valuable insights into the phylogenetic study of grass species. Moreover, more nuclear data will be necessary for further study on the intra- and inter-generic relationships of *Cleistogenes*.

## Data Availability Statement

The datasets presented in this study can be found in online repositories. The names of the repository/repositories and accession number(s) can be found below: https://www.ncbi.nlm. nih.gov/genbank/, MW194080; https://www.ncbi.nlm.nih.gov/genbank/, MW194081; https://www.ncbi.nlm.nih.gov/genbank/, MW194082; https://www.ncbi.nlm.nih.gov/genbank/, MW1940 83; https://www.ncbi.nlm.nih.gov/genbank/, MW194084; https://www.ncbi.nlm.nih.gov/genbank/, MW194085; https://www.ncbi. nlm.nih.gov/genbank/, MW194086; https://www.ncbi.nlm.nih. gov/genbank/, MW194087; https://www.ncbi.nlm.nih.gov/genbank/, MW194088; https://www.ncbi.nlm.nih.gov/genbank/, MW194089; https://www.ncbi.nlm.nih.gov/genbank/, MW1940 90; https://www.ncbi.nlm.nih.gov/genbank/, MW194091; https://www.ncbi.nlm.nih.gov/genbank/, MW194092; https://www.ncbi. nlm.nih.gov/genbank/, MW194093; and https://www.ncbi.nlm.nih. gov/genbank/, MW194094.

## Author Contributions

X-JZ, X-JQ, and S-JF designed the experiments. RW and KL carried out the experiment and analyzed the data. RW wrote the first draft of the manuscript. X-JZ, W-LC, X-JQ, and S-JF supervised and completed the writing. All authors collected the samples, revised, and approved the final manuscript.

## Conflict of Interest

The authors declare that the research was conducted in the absence of any commercial or financial relationships that could be construed as a potential conflict of interest.

## Footnotes

1 http://www.theplantlist.org/

2 http://bibiserv.techfak.uni-bielefeld.de/reputer/

3 http://sourceforge.net/projects/codonw/

## Supplementary Material

Supplementary Figure 1 The ML phylogeny of *Cleistogenes* and its closely related genera based on protein-coding genes.

Supplementary Figure 2 The BI phylogeny of *Cleistogenes* and its closely related genera based on protein-coding genes.

Supplementary Figure 3 The ML phylogeny of *Cleistogenes* and its closely related genera based on non-coding regions.

Supplementary Figure 4 The BI phylogeny of *Cleistogenes* and its closely related genera based on non-coding regions.

Supplementary Figure 5 The ML phylogeny of 78 chloridoid grasses based on complete plastomes.

Supplementary Figure 6 The BI phylogeny of 78 chloridoid grasses based on complete plastomes.

Supplementary Table 1 List of genes annotated in 12 *Cleistogenes* plastomes.

Supplementary Table 2 Types of SSRs.

Supplementary Table 3 Results of codon usage analysis.

Supplementary Table 4 Percentage of each amino acid.

Supplementary Table 5 Estimated divergence time for the *Ael*-*Cle*-*Ori*-*Tri* clade.
