# Supplementary figures and images for: Comparative Plastomes and Phylogenetic Analysis of Cleistogenes and Closely Related Genera (Poaceae)
Source: Front Plant Sci. 2021 Mar 25;12:638597. doi: 10.3389/fpls.2021.638597 (PMC8030268; doi:10.3389/fpls.2021.638597)

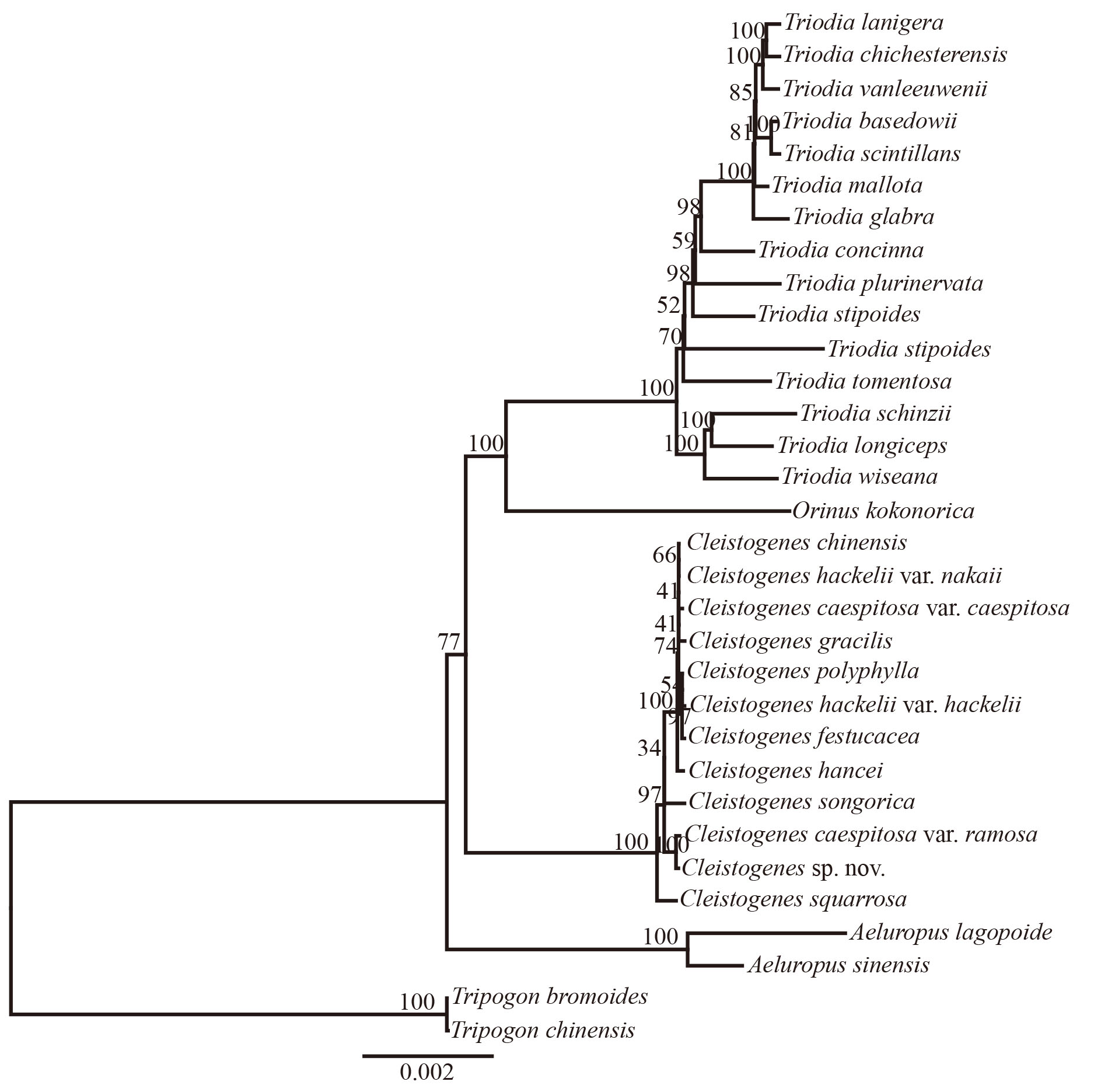

Supplement: Supplementary Figure 1 — The ML phylogeny of Cleistogenes and its closely related genera based on protein-coding genes. [file Image_1.JPEG]

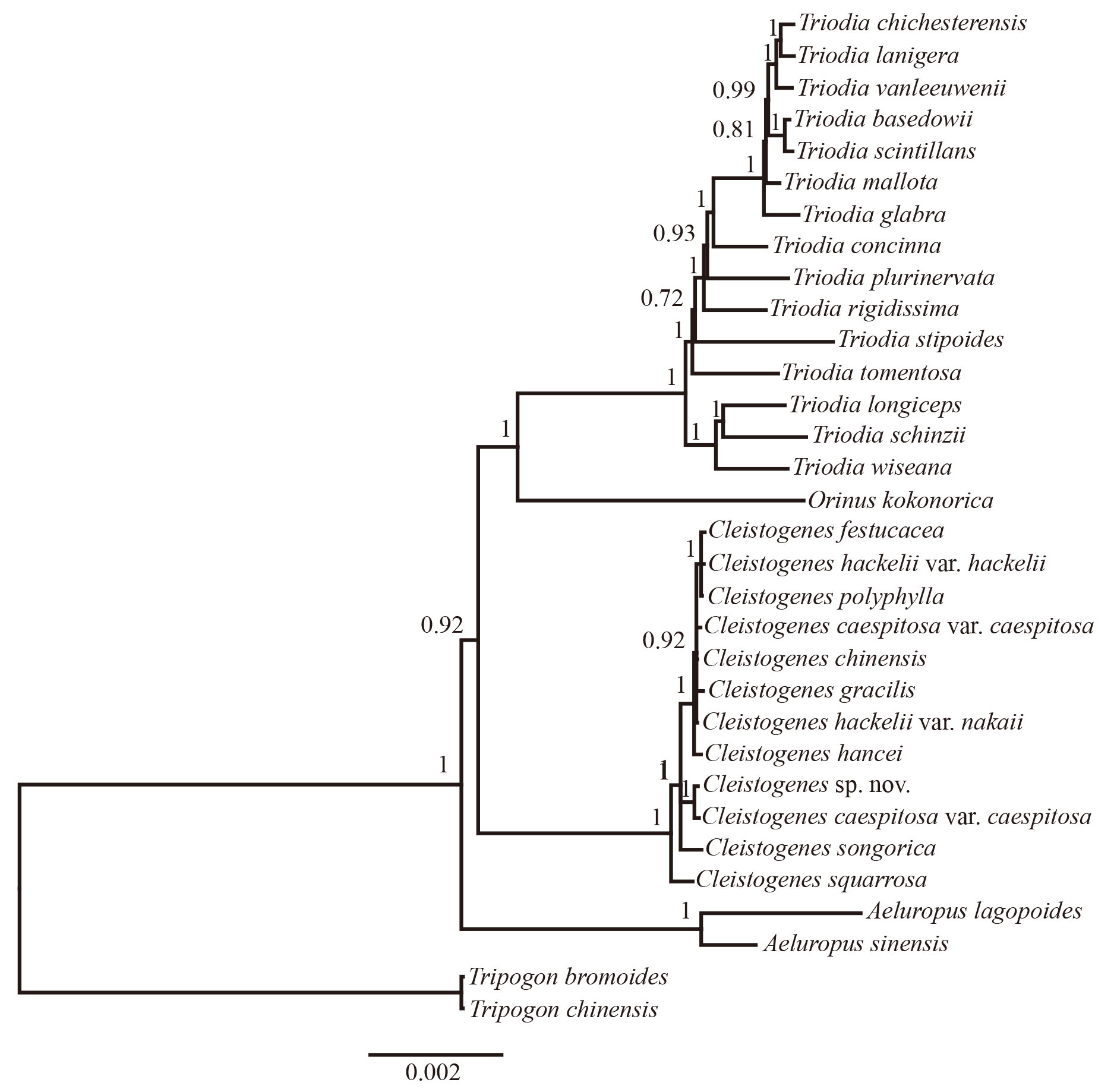

Supplement: Supplementary Figure 2 — The BI phylogeny of Cleistogenes and its closely related genera based on protein-coding genes. [file Image_2.JPEG]

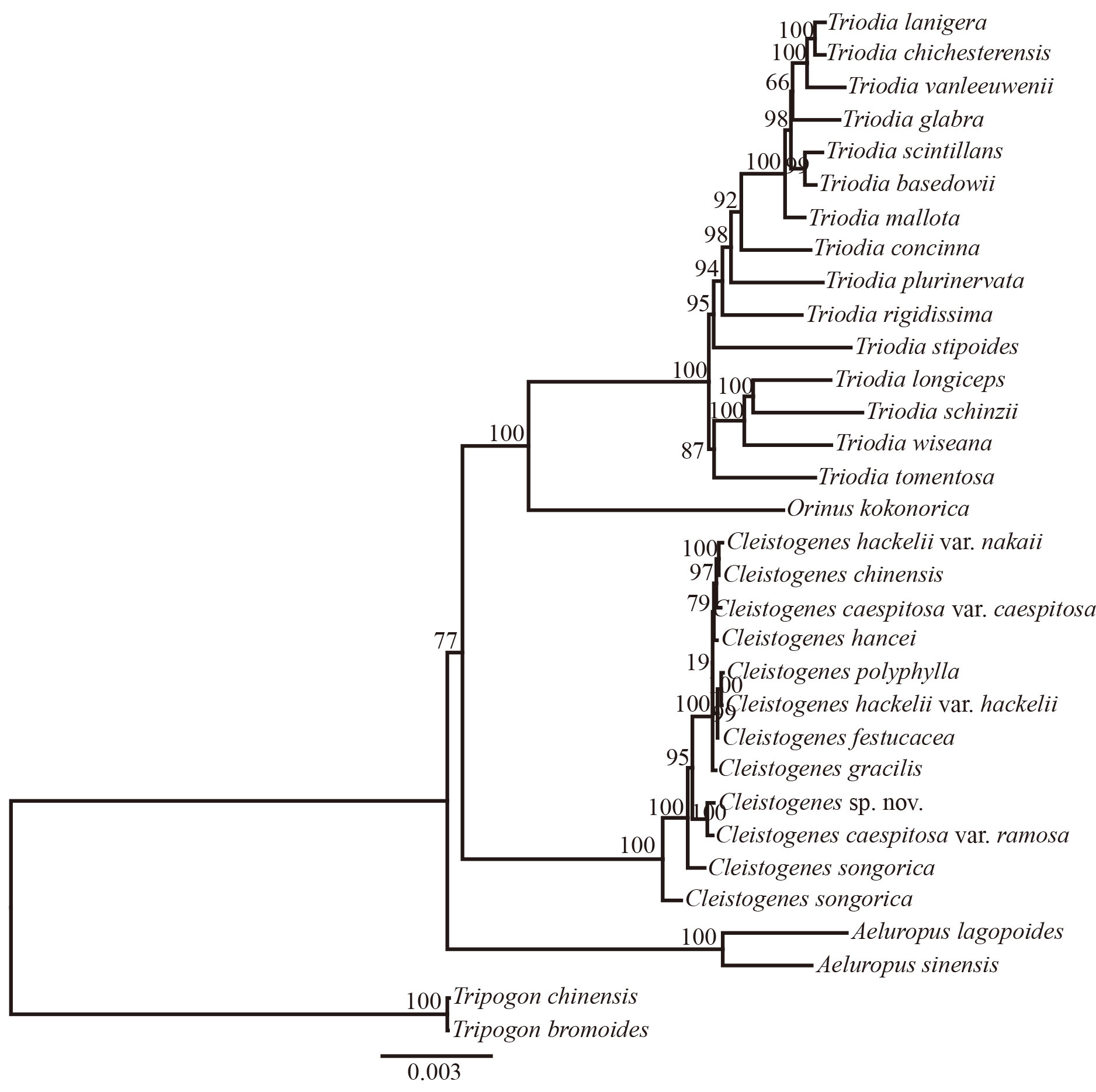

Supplement: Supplementary Figure 3 — The ML phylogeny of Cleistogenes and its closely related genera based on non-coding regions. [file Image_3.JPEG]

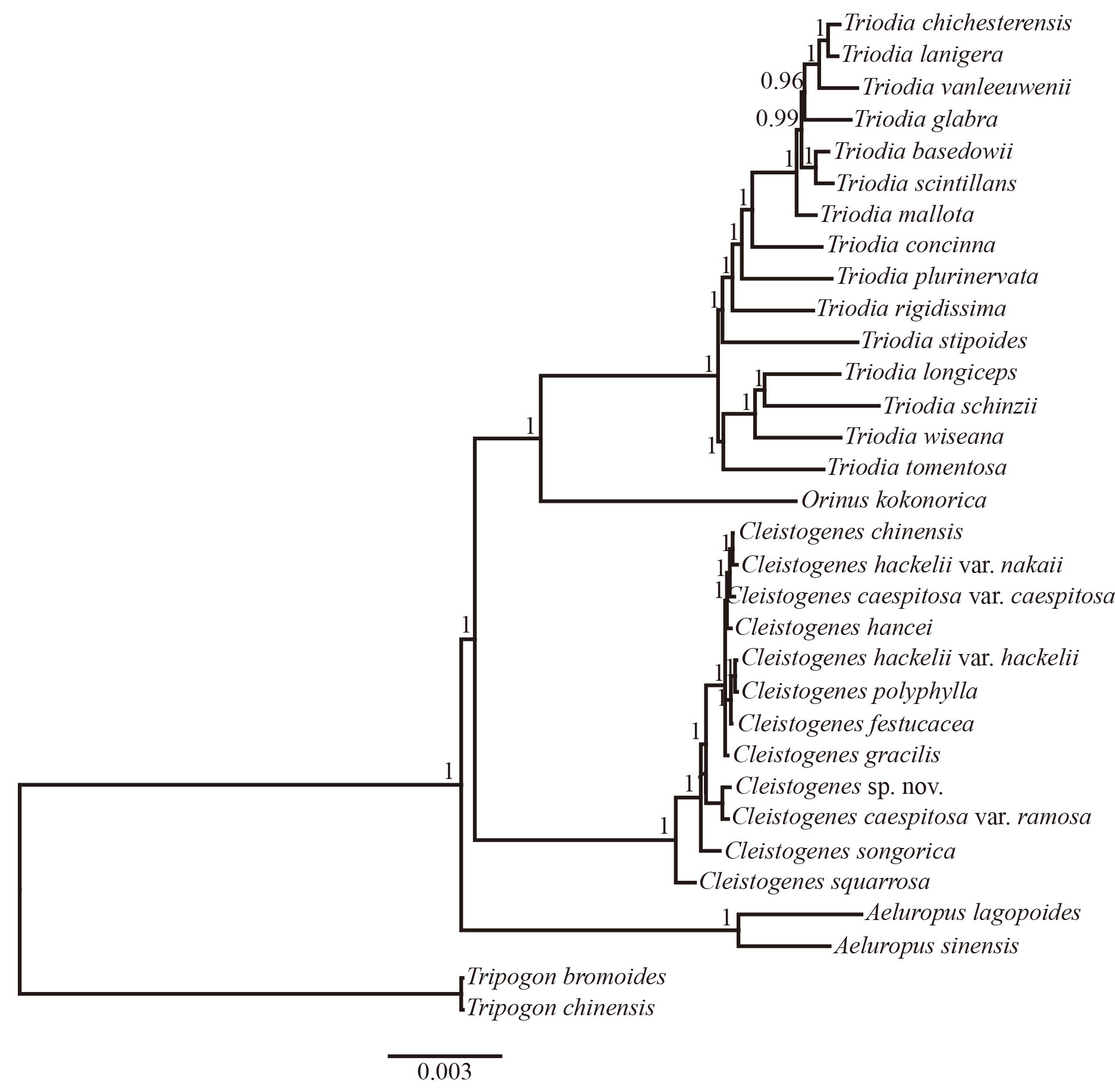

Supplement: Supplementary Figure 4 — The BI phylogeny of Cleistogenes and its closely related genera based on non-coding regions. [file Image_4.JPEG]

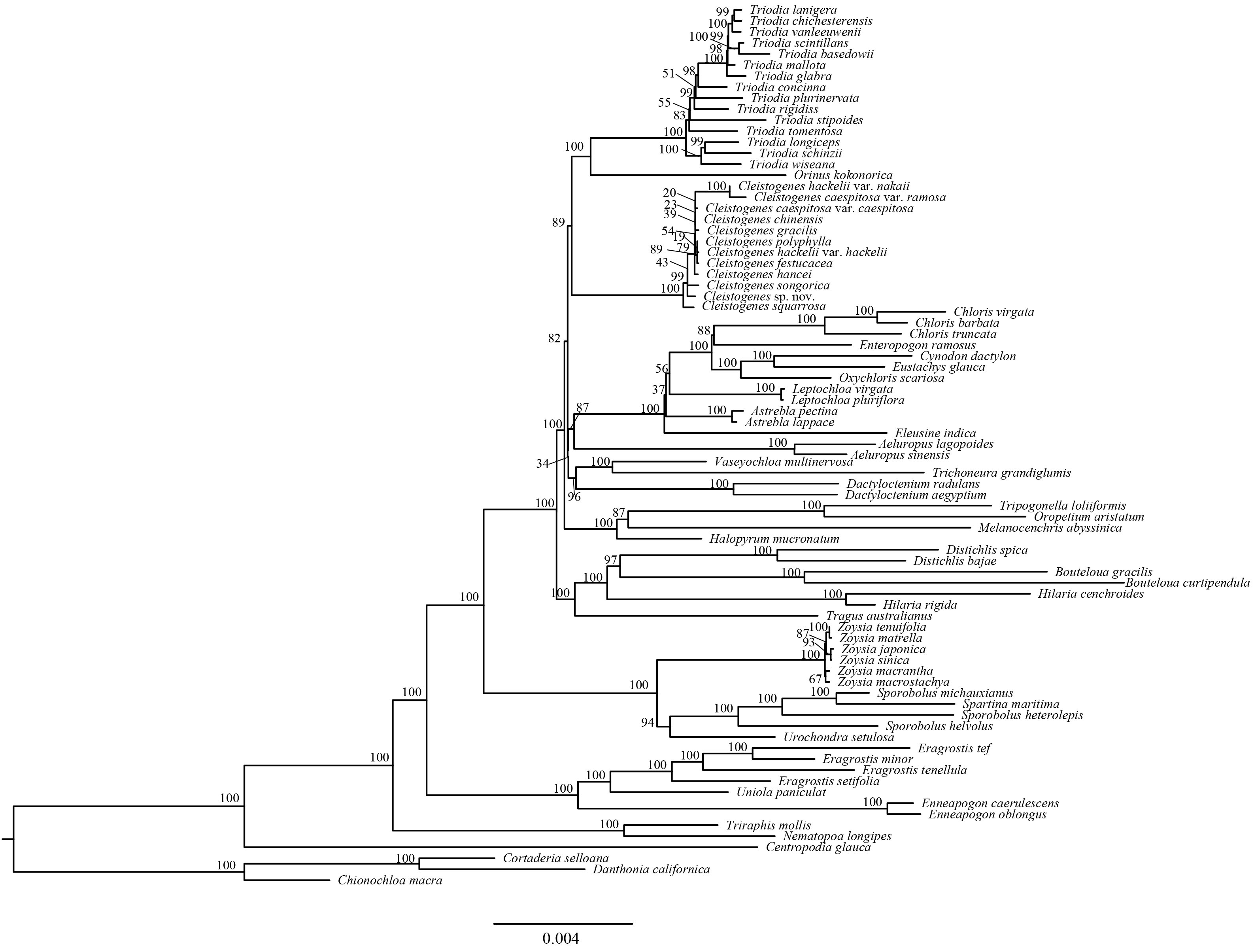

Supplement: Supplementary Figure 5 — The ML phylogeny of 78 chloridoid grasses based on complete plastomes. [file Image_5.JPEG]

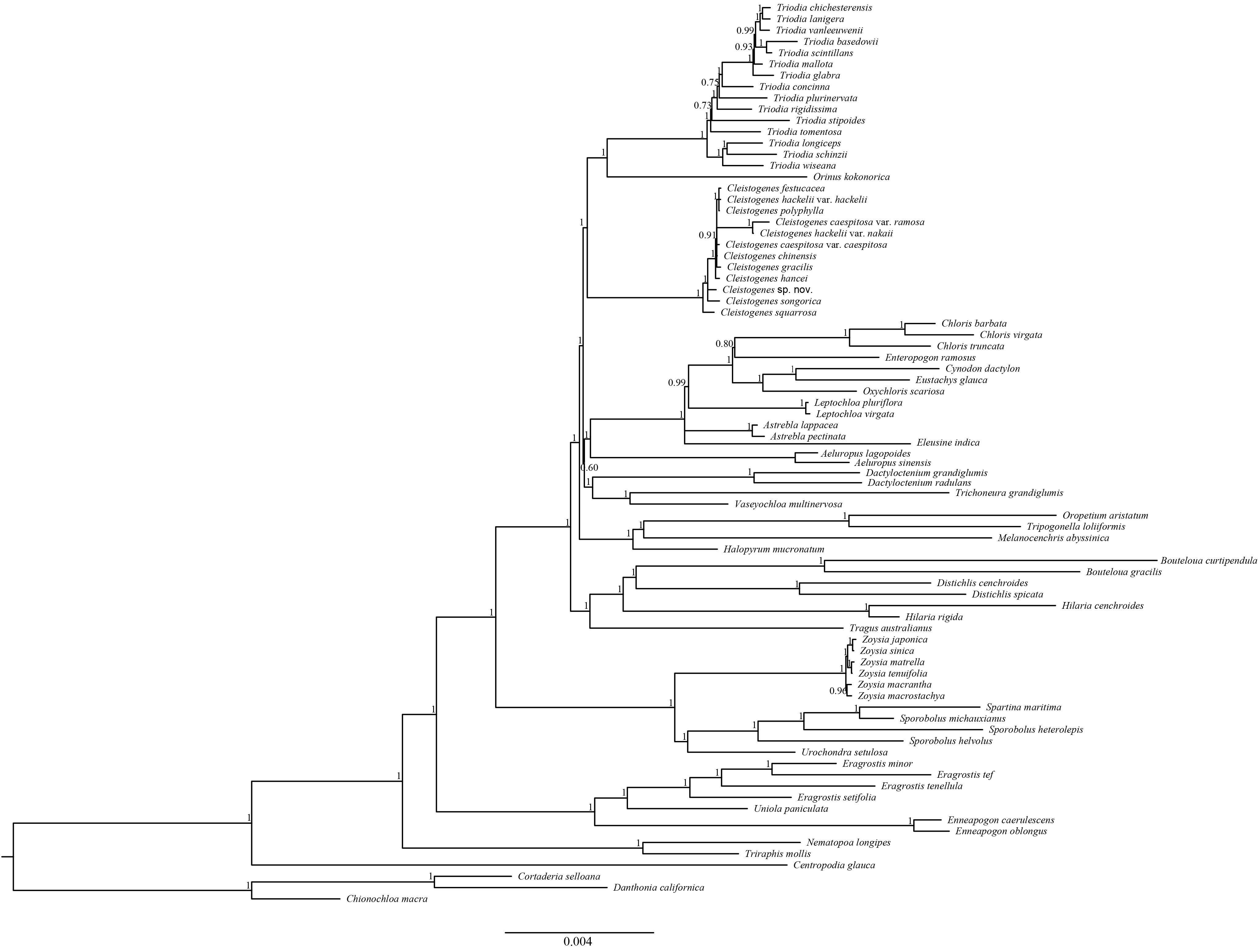

Supplement: Supplementary Figure 6 — The BI phylogeny of 78 chloridoid grasses based on complete plastomes. [file Image_6.JPEG]
