# Supplementary material for: Comparative Plastomes and Phylogenetic Analysis of Cleistogenes and Closely Related Genera (Poaceae)
Source: Front Plant Sci. 2021 Mar 25;12:638597. doi: 10.3389/fpls.2021.638597 (PMC8030268; doi:10.3389/fpls.2021.638597)
Supplement: Supplementary Table 1 — List of genes annotated in 12 Cleistogenes plastomes. [file Table_1.DOCX]

**Table S1.** List of genes annotated in 12 *Cleistogenes* plastomes

| **Gene category** | **Gene type** | **Gene** |
| --- | --- | --- |
| Photosynthesis | Subunits of photosystem I | *psaA*, *psaB*, *psaC*, *psaI*, *psaJ* |
|  | Subunits of photosystem II | *psbA*, *psbB*, *psbC*, *psbD*, *psbE*, *psbF*, *psbH*, *psbI*, *psbJ*, *psbK*, *psbL*, *psbM*, *psbN*, *psbT*, *psbZ* |
|  | Subunits of NADH dehydrogenase | *ndhA*, *ndhB*, *ndhC*, *ndhD*, *ndhE*, *ndhF*, *ndhG*, *ndhH*, *ndhI*, *ndhJ*, *ndhK* |
|  | Subunits of ATP synthase | *atpA*, *atpB*, *atpE*, *atpF*, *atpH*, *atpI* |
|  | Subunits of cytochrome b/f complex | *petA*, *petB*, *petD*, *petG*, *petL*, *petN* |
|  | Large subunit of rubisco | *rbcL* |
| Self-replication | Proteins of large ribosomal subunit | *rpl2*, *rpl14*, *rpl16*, *rpl20*, *rpl22*, *rpl23*, *rpl32*, *rpl33*, *rpl36* |
|  | Proteins of small ribosomal subunit | *rps2*, *rps3*, *rps4*, *rps7*, *rps8*, *rps11*, *rps12*, *rps14*, *rps15*, *rps16*, *rps18*, *rps19* |
|  | Subunits of RNA polymerase | *rpoA*, *rpoB*, *rpoC1*, *rpoC2* |
|  | Ribosomal RNAs | *rrn23*, *rrn16*, *rrn5*, *rrn4.5* |
|  | Transfer RNAs | *trnA-UGC*, *trnC-GCA*, *trnD-GUC*, *trnE-UUC*, *trnF-GAA*, *trnfM-CAU*, *trnG-GCC*, *trnG-UCC*, *trnH-GUG*, *trnI-CAU*, *trnI-GAU*, *trnK-UUU*, *trnL-CAA*, *trnL-UAA*, *trnL-UAG*, *trnM-CAU*, *trnN-GUU*, *trnP-UGG*, *trnQ-UUG*, *trnR-ACG*, *trnR-UCU*, *trnS-GCU*, *trnS-GGA*, *trnS-UGA*, *trnT-GGU*, *trnT-UGU*, *trnV-GAC*, *trnV-UAC*, *trnW-CCA*, *trnY-GUA* |
| Biosynthesis | Maturase | *matK* |
|  | Protease | *clpP* |
|  | Envelope membrane protein | *cemA* |
|  | c-type cytochrome synthesis gene | *ccsA* |
|  | Translation initiation factor | *infA* |
| Unknown function | Conserved hypothetical chloroplast Reading Frames | *ycf2*, *ycf3*, *ycf4* |
